# METTL16 suppresses ferroptosis in cholangiocarcinoma by promoting ATF4 via m^6^A modification

**DOI:** 10.7150/ijbs.97886

**Published:** 2025-01-01

**Authors:** Senfeng Zhao, Jiahui Cao, Ruopeng Liang, Tingting Peng, Shitao Wu, Zhipu Liu, Yahui Wu, Liming Song, Chenguang Sun, Yin Liu, Junmou Gu, Libo Wang, Rongtao Zhu, Weijie Wang, Yuling Sun

**Affiliations:** 1 Department of Hepatobiliary and Pancreatic Surgery, The First Affiliated Hospital of Zhengzhou University, Zhengzhou, China.; 2 Institute of Hepatobiliary and Pancreatic Diseases, Zhengzhou University, Zhengzhou, China.; 3 Zhengzhou Basic and Clinical Key Laboratory of Hepatopancreatobiliary Diseases, Zhengzhou, China.; 4 Department of Neurology, The First Affiliated Hospital of Zhengzhou University, Zhengzhou, China.; 5 Department of Hepatobiliary and Pancreatic Surgery, Zhengzhou Central Hospital Affiliated to Zhengzhou University, Zhengzhou, China.

**Keywords:** m^6^A, ferroptosis, METTL16, ATF4, cholangiocarcinoma

## Abstract

**Background:** N6-methyladenosine (m^6^A) modification is the most common post-transcriptional modifications, which is critical for the metabolism of ferroptosis-related RNAs. Yet, the impact of m^6^A modification on ferroptosis in cholangiocarcinoma (CC) is far from clear.

**Methods:** Public databases and tissue arrays were applied to explore the clinical relevance of METTL16 in CC. Then, the effects of METTL16 on growth and ferroptosis were studied *in vitro* and *in vivo*. Mechanistically, RNA-sequencing, methylated RNA immunoprecipitation, dual-luciferase reporter assays and RNA stability assays were used to identify the METTL16/ATF4 axis in ferroptosis in CC.

**Results:** Clinically, we find that METTL16 is overexpressed and associated with a poor prognosis in patients with CC. Functionally, METTL16 protects against ferroptosis by maintaining mitochondrial homeostasis, including mitochondrial structure, membrane potential and energy products. It also decreases cellular metabolism of Fe^2+^ and lipid peroxide, thereby promoting cell growth *in vitro* and *in vivo*. Mechanistically, ATF4 is a novel target of METTL16 and METTL16 enhances the m6A level and expression of ATF4 mRNA by inhibiting its decay, which further prevented ferroptosis in CC via m6A modification.

**Conclusions:** Our findings highlighted the role of METTL16/ATF4 in ferroptosis, which sheds light on potential therapeutic strategies for CC.

## Introduction

Cholangiocarcinoma (CC) is a rare malignancy, accounting for less than 3% of gastrointestinal tumors, but its morbidity and mortality have been increasing worldwide in recent decades 1-3. Currently, surgery remains the preferred treatment for early-stage CC, and the outcomes doesn't come into satisfaction because of high tumor aggressiveness and recurrence. Despite the application of targeted agents combined with checkpoint inhibitors, advanced CC remains problematic due to drug resistance and high tumor heterogeneity 4, 5. Therefore, it is imperative to deepen the understand underlying occurrence and development of CC to improve future treatment outcomes.

N6-methyladenosine (m^6^A), which was initially identified in 1974, is the predominant post-transcriptional modification of RNA. It is involved in almost full processes of RNA metabolism, such as RNA processing, transport, translation and decay 6-8. To date, three classes of enzymes have been shown to keep this modification reversible and dynamic: methyltransferases, demethylases and reading proteins. Methyltransferases, which are exemplified by METTL3, METTL14, METTL16 and WTAP, cause RNA methylation. In contrast, demethylases including ALKBH5 and FTO remove methylation from RNA. In addition, m^6^A motifs are recognized and bound by reading proteins, such as YTHDF/2/3, IGF2BP1/2/3 and YTHDC1/2 8, 9. Previous researches have revealed that m^6^A modification have great effect on various cellular processes, including proliferation, differentiation and death 10, 11. Intriguingly, resistance to cell death is a characteristic hallmark of tumors, facilitating cancer progression and acquired resistance to anti-tumor therapies 12. However, as comparatively few studies on this topic, less is known about m6A in cell death of CC.

Ferroptosis is a unique programmed cell death distinguished by the accumulation of Fe^2+^ and lipid peroxidation (LPO) 13. Morphologically, ferroptosis is characterized by mitochondrial shrinkage, with a decrease or vanishing of mitochondrial cristae and a rise in mitochondrial membrane density 14, 15. Increasing studies have highlighted the the vital importance of ferroptosis in the tumorigenesis of cancers, such as colon cancer, live cancer, and pancreatic cancer 16-18. Therefore, novel pharmacological strategies targeting ferroptosis have emerged as therapeutic interventions. Agents like sorafenib and sulfasalazine have been used in the treatment of hepatocellular carcinoma by inducing ferroptosis 19, 20. Furthermore, ATF4 plays a vital role in this process. ATF4, a transcription factor, activates SLC7A11 during oxidative stress, contributing to the transport of extracellular cystine and the production of glutathione (GSH), thereby preventing ferroptosis 21, 22. It also promotes the expression of HSPA5, which binds to GPX4 to arrest its degradation and subsequent lipid peroxidation, further protecting against ferroptosis 23. Nevertheless, ATF4-mediated ferroptosis in CC remains incompletely understood.

Here, we analyzed the expression of m^6^A-associated genes in CC and explored the underlying mechanisms. Clinically, the m^6^A methyltransferase METTL16 was upregulated and acted as an independent prognostic factor of CC. Mechanistically, METTL16 regulated the migration and proliferation of CC by suppressing ferroptosis through ATF4 in an m^6^A-dependent manner. In total, we highlighted the importance of METTL16-mediated m^6^A modification and ferroptosis in CC and provided a promising strategy for the treatment of CC.

## Materials and Methods

### Cell culture and transfection

The human extrahepatic CC cell line KMBC and intrahepatic CC cell line RBE were purchased from the Cell Bank of Chinese Academy of Sciences (Shanghai, China), and cultured in RPMI-1640 (Gibco, Invitrogen, Carlsbad, USA) supplemented with 10% fetal bovine serum (Life-iLab, Shanghai, China). siRNAs (si METTL16, si ATF4 and paired control) came from RiboBio (Guangzhou, China) and the sequences showed in Table S1. Plasmids (sh METTL16, METTL16, ATF4 and paired control) came from GenePharma (Shanghai, China). Lipofectamine^TM^ 2000 (Invitrogen, Carlsbad, USA) was used for transient transfection.

### Cell counting kit-8 (CCK8) assay

As to intervention by erastin (MCE, Shanghai, China) or RSL3 (MCE, Shanghai, China), 5 × 10^3^ normal cells were cultured in 96-well plates overnight. Then, cells were treated with RSL3 or erastin for 24 h and added 10 μL CCK8 reagent (TargetMol, Shanghai, China). As to intervention by liproxstatin or ferrostatin-1, 5 × 10^3^ transfected cells were cultured in 96-well plates overnight. Then, cells were treated with liproxstatin (MCE, Shanghai, China) or ferrostatin-1 (MCE, Shanghai, China) and added 10 μL CCK8 reagent at 12-hour intervals until 48 hours. After incubation for 2 h, the cell viability was measured.

### EdU and colony formation assays

The EdU and colony formation assays were used to assess cell proliferative capacity. As to EdU assay, 1 ×10^4^ transfected cells were plated in 24-well plates and cultured overnight. The proportion of proliferative cells was evaluated by EdU Cell Proliferation Image Kit (Abbkine, Wuhan, China), based on images captured by fluorescence microscopy. For colony formation, 1500 transfected cells were cultured in 6-well plates for 2 weeks. The colonies were fixed with 4% paraformaldehyde and stained with 0.1% crystal violet (Solarbio, Beijing, China).

### Transwell and wound-healing assays

The Transwell and wound-healing assays were applied to evaluate cell migration ability. For Transwell assay, the upper chambers were cultured with 2 × 10^4^ transfected cells in serum-free medium, and the chambers below were filled with 20% FBS medium. 24 h later, the upper chambers were fixed with 4% paraformaldehyde and stained with 0.1% crystal violet. For wound-healing assay, 2.0 × 10^5^ transfected cells were cultured in 12-well plates until to complete confluence. Finally, a scratch was made, and the healing situation was recorded after 0 and 24 h.

### Apoptosis

Annexin V/PI apoptosis kit (UElandy, Suzhou, China) were used to assess the cell apoptosis. Briefly, cells in 6-cm dishes were transfected for 48h. According to the manufacturer's instructions, cells were resuspended in staining buffer, and tested by flow cytometry.

### Western blotting (WB)

Proteins were obtained from tissues and cells by RIPA buffer (Solarbio, Beijing, China) with protease inhibitors (Beyotime, Shanghai, China). The total protein concentration was determined using a BCA protein assay kit (EpiZyme, Shanghai, China), 20 μg protein were separated by SDS-PAGE and transferred to 0.45 μm PVDF membranes (Millipore, Billerica, USA). Then, the membranes were blocked with 5% non-fat milk at room temperature for 2 h and incubated with primary antibodies at 4°C overnight. Next day, the membranes were incubated with secondary antibodies specific to the primary antibodies at room temperature for 1 h. The visualization of the bound antibodies was achieved using an ECL (Enhanced Chemiluminescence) reagent (Millipore, Billerica, USA). The primary antibodies and secondary antibodies used are detailed in Table S2.

### Quantitative reverse transcription PCR (qPCR)

Total RNA was extracted from tissues and cells by TRIzol (CWBIO, Beijing, China), followed by cDNA synthesis with SweScript All-in-One RT SuperMix (Servicebio, Wuhan, China). Subsequently, qPCR was conducted with SYBR Green qPCR Master Mix (Servicebio, Wuhan, China). The specific primers employed for qPCR are detailed in Table S3.

### RNA-sequencing

RNA-sequencing services were provided by Kaitaibio (Hangzhou, China). mRNA was enriched from total RNA, and an RNA library was constructed. After the library passed the quality inspection, RNA-sequencing was conducted on an Illumina NovaSeq 6000. The raw data were normalized and analyzed by bioinformatics.

### Methylated RNA immunoprecipitation (MeRIP)

Total RNA was obtained by TRIzol, and RNA immunoprecipitation assays were conducted using a Magna RIP Kit (Millipore, Darmstadt, Germany). Using an anti-m^6^A antibody and normal rabbit IgG to immunoprecipitated RNA-binding proteins. The enriched RNA was released, purified, and eluted by RNA Clean & Concentrator ^TM^ (Zymo Research, Irvine, USA). qPCR was performed following MeRIP to quantify changes in the m^6^A methylation level of target genes. Furthermore, nucleic acid electrophoresis was used to verify the target gene in cDNA from MeRIP-qPCR.

### Dual-luciferase reporter assay

ATF4 mRNA fragments (CDS, from 341-488) containing wild-type and mutant m^6^A motifs (the second predicted site, A was replaced by C) were directly synthesized and used to construct luciferase reporter plasmids by GeneChem (Shanghai, China). Wild-type or mutant ATF4 luciferase plasmids were co-transfected with siMETTL16 and controls. Luciferase activity was quantified by a dual-luciferase reporter assay system (Vazyme, Nanjing, China). The relative activity of the firefly luciferase was standardized by referencing it against the activity of renilla luciferase.

### RNA stability assay

For RNA stability assays, cells were transfected with si METTL16, METTL16 plasmids and controls. Then, the cells were exposed to 10 μg/ml actinomycin D (Med Chem Express, New Jersey, USA) for 0 h, 2 h, 4 h, and 8 h. Subsequently, total RNA was extracted, and the ATF4 mRNA levels were quantified by qPCR.

### Superoxide dismutase (SOD), and antioxidant capacity assays

To determine the SOD and antioxidant capacity levels in cells, Total Superoxide Dismutase Assay Kit with WST-8 (Beyotime, Shanghai, China) and Total antioxidant capacity assay kit (Nanjing Jiancheng Bioengineering Institute, Nanjing, China) were used, respectively. Briefly, cells in 6-cm dishes were processed following the manufacturer's protocol, and the absorbance of the reaction mixture was measured at 450 nm and 405 nm, respectively.

### GSH, malondialdehyde (MDA) and ATP assays

To measure GSH, MDA and ATP levels in cells, GSH assay kit (Nanjing Jiancheng Bioengineering Institute, Nanjing, China), Lipid Peroxidation MDA Assay Kit (Beyotime, Shanghai, China) and ATP assay kit (Beyotime, Shanghai, China) were used, respectively. In short, cells in 10-cm dishes or tumor tissues from xenografts were lysed with Cell lysis buffer for Western and IP (Beyotime, Shanghai, China) and subsequently analyzed following the manufacturer's instruction. Finally, the absorbance at 532 or 420 nm and the fluorescence intensity were measured.

### Mitochondrial membrane potential (MMP), reactive oxygen species (ROS), superoxide anion (SOA), Fe^2+^ and LPO assays

TMRE (Beyotime, Shanghai, China), DCFH-DA (Beyotime, Shanghai, China), Dihydroethidium (Beyotime, Shanghai, China), Phen Green SK (GlpBio, Montclair, CA, USA) and BODIPY™ 581/591 C11 (Thermo Fisher Scientific, Waltham, USA) were used to assess the MMP, ROS, SOA, Fe^2+^ and LPO levels in cells, respectively. Briefly, cells in 6-cm dishes were incubated with 10 μM TMRE, 10 μM DCFH-DA, 5 μM Dihydroethidium, 5 μM Phen Green SK and 10 μM BODIPY 581/591 C11 for 30 min. Finally, fluorescence microscopy was used to analyze the MMP, Fe^2+^, ROS and LPO levels in cells, and flow cytometry were used to analyze the SOA and LPO levels.

### Transmission electron microscopy (TEM)

Cells were fixed using 2.5% glutaraldehyde in 0.1 mM phosphate buffer, and incubated in 1% OsO_4_ for 2 h. Once dehydrated, the cells were embedded in epoxy resin. After resin blocks trimmed, Ultrathin sections were made using an ultramicrotome and stained with lead citrate and uranyl acetate. Finally, the stained ultrathin sections were observed using TEM.

### Confocal laser scanning microscopy (CLSM)

CLSM was employed to confirm the location of METTL16 in cells. The cells were fixed with 4% paraformaldehyde for 45 min, permeabilized using 0.3% Triton X-100 for 30 min, and blocked with 10% goat serum for 1 h. Next, the cells were incubated overnight at 4°C with an anti-METTL16 antibody, followed by incubation with goat anti-rabbit IgG fluorescent antibody for 1 h at 37°C. To visualize the cell nuclei, the cells were stained with DAPI (Solarbio, Beijing, China). Finally, the cells were examined using a Zeiss LSM880 CLSM with a 100× oil immersion objective.

### Animal models

Animal experiments were approved by Animal Ethics Committee of Zhengzhou University. For xenograft models, female BALB/c nude mice (5 weeks, Beijing HFK Bioscience Co., Ltd., Beijing, China) were used to confirm tumor growth, metastasis and ferroptosis* in vivo*. KMBC cells (1 × 10^7^ cells) were percutaneously injected into the axilla of mice. After two weeks, the tumors were dissected and divided into 50 cm^3^ masses. Then, the tumor masses were subcutaneously implanted into the axilla of mice and subsequently injected with sh METTL16, METTL16 and control plasmids along with Lipo8000^TM^ Transfection Reagent (Beyotime, Shanghai, China) every three days to establish METTL16-knockdown and METTL16-overexpressing subcutaneous xenografts. The body weight of the mice and the size of the tumors were measured every three days until the mice were sacrificed. Dissected tumor tissues were preserved at -80°C or 10% formalin fixation and used for later experiments.

### Immunohistochemistry (IHC) and hematoxylin eosin (HE) staining

Xenografts were embedded in paraffin and further precisely sliced into 4-μm-thick sections for HE and IHC staining. For HE, the sections were counterstained with hematoxylin (Servicebio, Wuhan, China), followed by incubation in DAB substrate solution (Servicebio, Wuhan, Chinas). For IHC, sodium citrate buffer was used for antigen retrieval. Then, the sections were incubated with 3% hydrogen peroxide for 20 min and blocked with 3% BSA for 1 h at room temperature. Then, the sections were incubated with primary antibodies overnight at 4°C. Following this, a secondary antibody was added and incubated for 60 min at room temperature. All the sections were observed by microscopy.

### Bioinformatics analysis

Three gene expression profiles, namely, GSE132305 (extrahepatic CC), GSE107943 (intrahepatic CC) and GSE76297 (only use intrahepatic CC) were obtained from the GEO. Differentially expressed genes were screened with the Limma package. GO and KEGG were used for functional enrichment analysis. The expression of METTL16 was analyzed by DepMap. The proteins level of METTL16 and ATF4 was studied by the Human Protein Atlas (HPA). SRAMP was used to predict m^6^A modification sites on ATF4 mRNA.

### Statistical analysis

All the experiments were independently conducted three times, and the data were analyzed by GraphPad Prism 8. The results are presented as the mean ± standard deviation (SD). The t tests or two-way ANOVA was used to analyze differences in normally distributed data between two groups. The Kaplan-Meier method was employed for survival analysis. Pearson's correlation test was used to assess the correlation between METTL16 expression and ATF4 expression. *p<0.05* was considered significant.

## Results

### METTL16 is overexpressed in CC and serves as a poor prognostic factor

**Figure 1:**
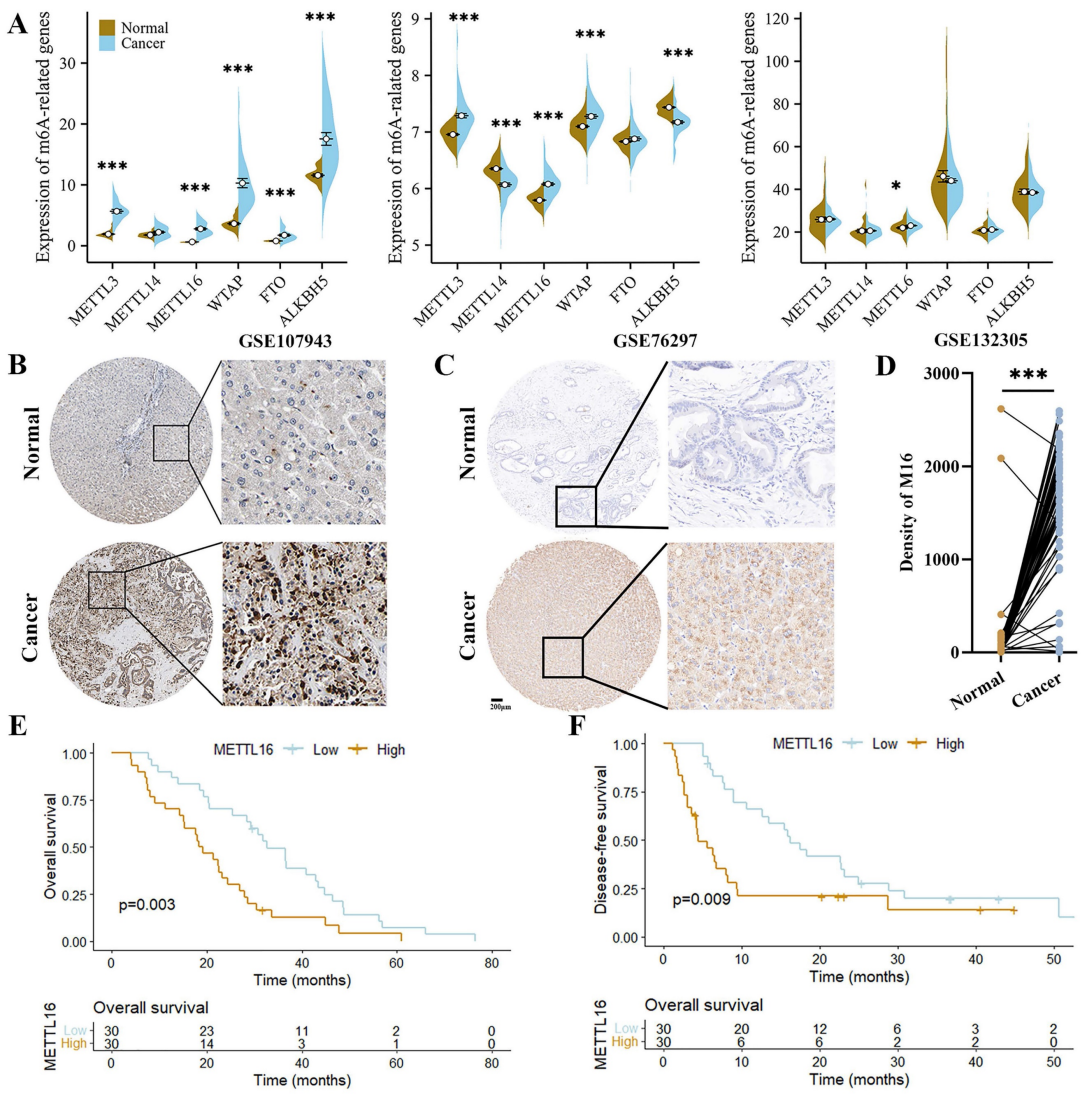
** METTL16 (M16) is upregulated in CC.** (A) The expression of m6A-related genes from GSE107943, GSE76297 and GSE132305. (B) The images of IHC staining for M16 in CC and normal tissues from HPA. (C-D) The protein level of M16 in the tissue array of CC was examined by IHC (n=60). (E-F) Kaplan-Meier analysis of the OS (E) and DFS (F) of CC patients from the tissue array. The data are presented as the mean ± SD. * p < 0.05, *** p < 0.005.

Initially, we queried the expression of the most important m^6^A regulatory genes in GEO datasets to explore the role of m^6^A modifications in CC. As shown, *METTL3*, *METTL16* and *WTAP* were significantly upregulated in CC tissues (2/3 datasets), implying a vital effect of m^6^A modification on the progression of CC. Among these genes, only *METTL16* was elevated in all datasets (Figure 1A). Subsequently, the clinical value of METTL16 was further analyzed with HPA and CC tissue array (60 CC tissues and paired adjacent normal tissues). We found that METTL16 expression was significantly higher in the cancer tissues (Figure 1B-D). Moreover, high *METTL16* expression shorten the overall survival (OS) and disease-free survival (DFS) of CC patients (Figure 1E and F). Collectively, these findings suggest that METTL16 is overexpressed in CC and acts a risk factor for CC patient prognosis.

### METTL16 enhances tumorigenicity in CC

**Figure 2:**
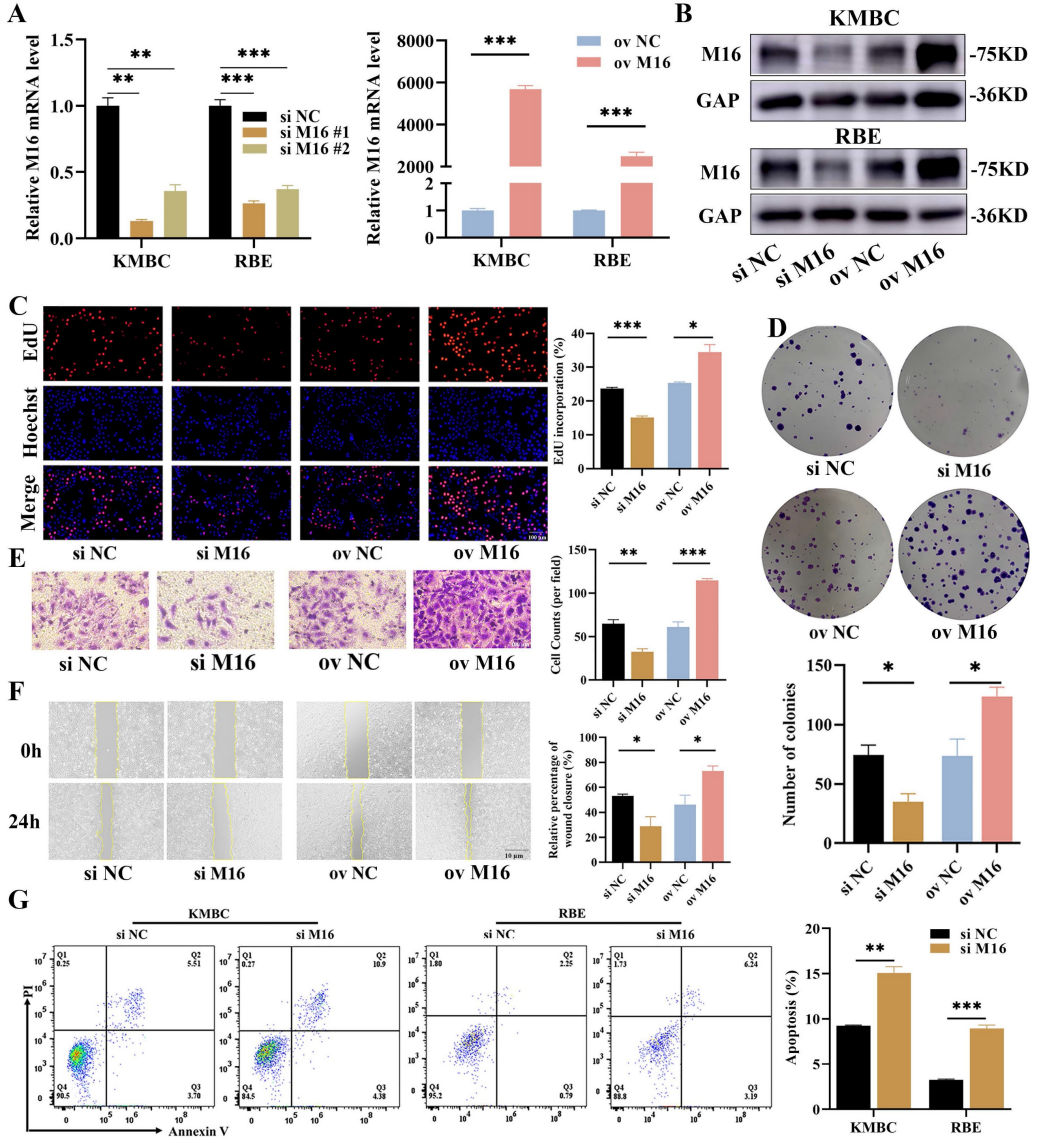
** M16 promotes CC progression.** (A-B) M16 knockdown or overexpression in KMBC and RBE cells was confirmed by qPCR (A) and WB (B). (C-F) The proliferation and migration of M16-knockdown or M16-overexpressing KMBC cells were investigated by EdU assays (C), colony formation assays (D), Transwell assays (E) and wound-healing assays (F). (G) the apoptosis in M16-knockdown KMBC and RBE cells were measured by flow cytometry. The data are presented as the mean ± SD. GAP: GAPDH. * p < 0.05, ** p < 0.01, *** p < 0.005.

Given that METTL16 is highly expressed in CC, we speculated that METTL16 may have pro-cancer effects in CC. We analyzed the the expression of METTL16 in CC cell lines by DepMap (Figure S1A), and constructed METLL16-knockdown and METLL16-overexpressing cells in KMBC and RBE by siRNAs and METTL16 overexpression plasmid to define the functions of METTL16 in CC (Figure 2A and B). CCK8 assay was used and showed that the survival was reduced after METTL16 knockdown (Figure S1B, #1 sequence of METTL16 was used after). We performed proliferative assays and found that proliferation ratio was much lower in METTL16-knockdown cells, while that was higher in METTL16-overexpressing cells (Figure 2C and D, Figure S1C and D). Then, Transwell and wound-healing assays were used to evaluate the cell migration and the results displayed that METTL16-knockdown cells exhibited slower migration, while METTL16-overexpressing cells exhibited the opposite trend (Figure 2E and F, Figure S1E and F). Next, the cell apoptosis assays were applied and showed that there is a lower apoptotic in METTL16-knockdown cells (Figure 2G). Overall, these results indicate that METTL16 promotes growth in CC *in vitro*.

### METTL16 inhibits oxidative stress in CC

**Figure 3:**
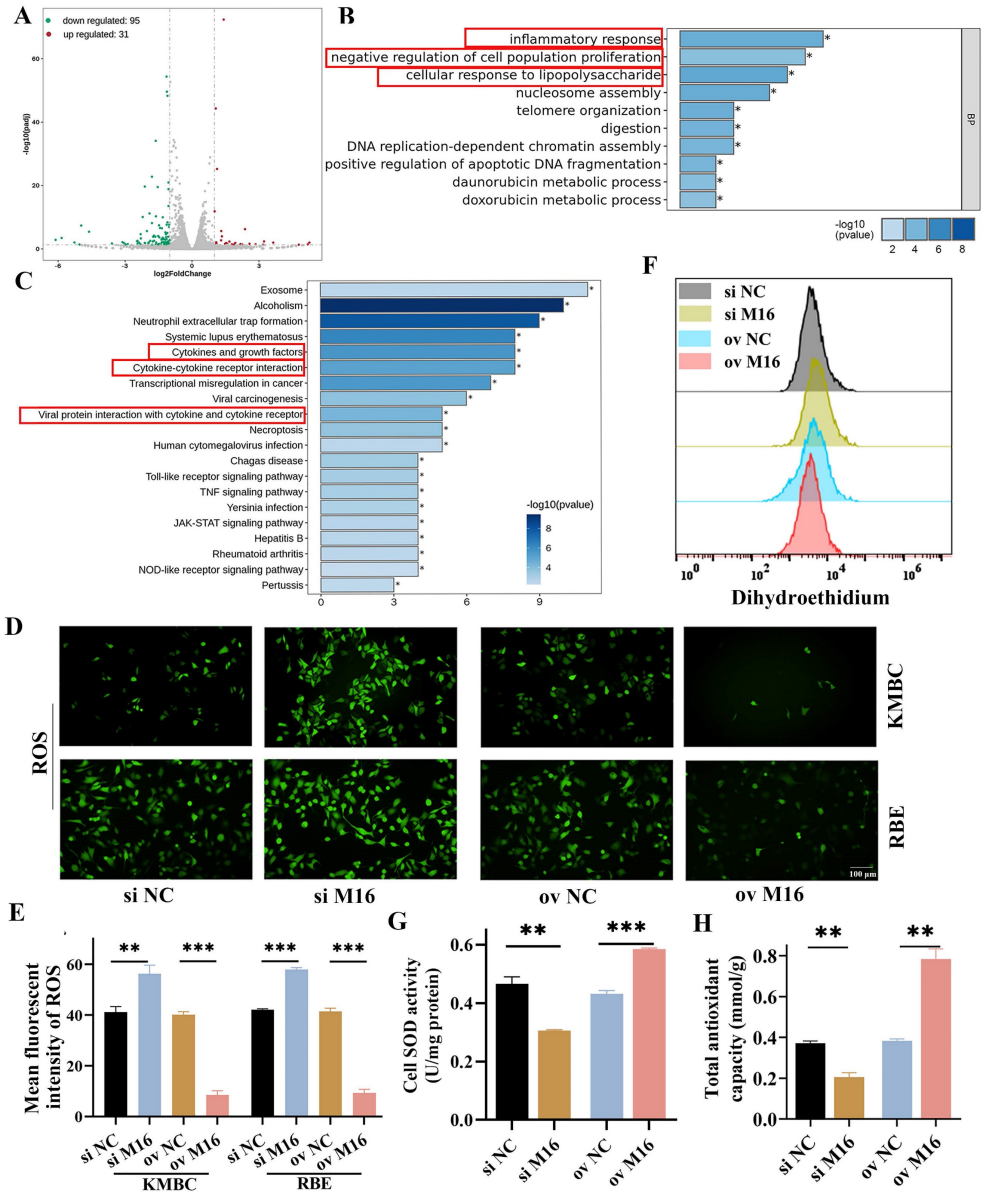
** M16 is closely related to oxidative stress.** (A) Volcano plot showing differential genes (|log2(fold change)|> 1 and p < 0.05) between M16-knockdown and control KMBC cells. (B-C) GO (B) and KEGG (C) analysis indicated that oxidative stress and cell proliferation were largely affected by M16 knockdown. (D-E) ROS levels in M16-knockdown or M16-overexpressing KMBC and RBE cells were assayed by fluorescence microscopy. (F) SOA levels in M16-knockdown or M16-overexpressing KMBC cells were assayed by flow cytometry. (G-H) SOD (G), total antioxidant capacity (H) levels in M16-knockdown or M16-overexpressing KMBC cells were measured. The data are presented as the mean ± SD. ** p < 0.01, ** p < 0.01, *** p < 0.005.

The results described above demonstrated that METTL16 is essential for the development of CC. However, the exact mechanism involved in this process remains unclear. To comprehensively explore the mechanism by which METTL16 regulates CC, RNA-sequencing was performed. We found 95 downregulated genes and 31 upregulated genes in METTL16-knockdown KMBC cells relative to control cells (Figure 3A). Then, these differential genes were analyzed by GO and KEGG and indicated that METTL16 has important effects on the oxidative stress and cell proliferation, such as the inflammatory response, cellular response to cytokine‒cytokine receptor interaction, lipopolysaccharide, negative regulation of cell population proliferation, cytokines and growth factors, and viral protein interaction with cytokines and cytokine receptors (Figure 3B and C). SOD, SOA, ROS and antioxidant capacity levels were main indicators of oxidative stress 24-27. Hence, we measured intracellular ROS, SOA, SOD and antioxidant capacity levels in KMBC or RBE cells. The results showed that METTL16 knockdown increased intracellular ROS and SOA but reduced SOD and antioxidant capacity levels, whereas METTL16 overexpression decreased intracellular ROS and SOA but elevated SOD and antioxidant capacity levels (Figure 3D-H). In sum, these findings show that METTL16 regulates CC through oxidative stress.

### METTTL16 inhibits ferroptosis in CC

**Figure 4:**
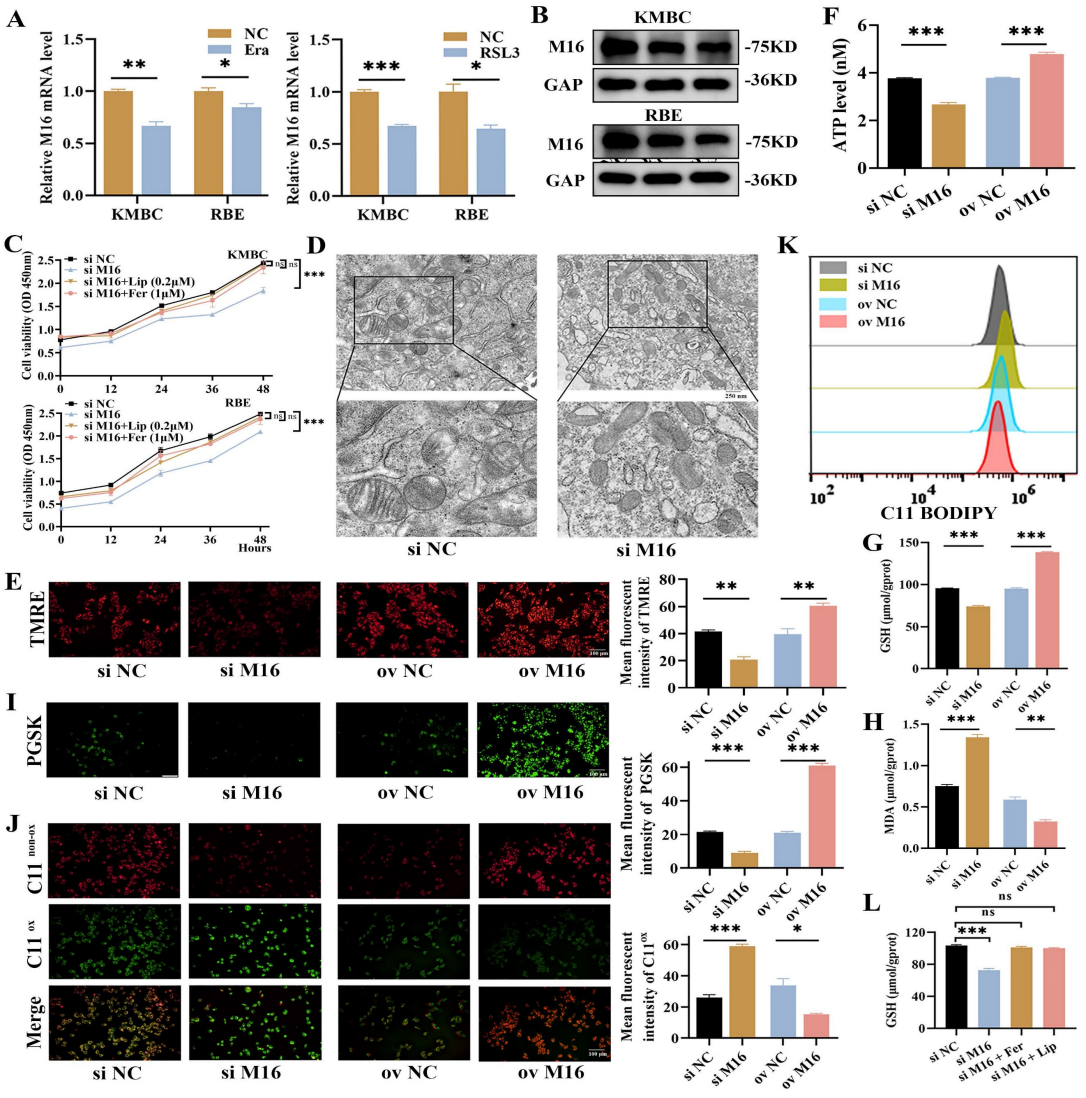
** M16 inhibits ferroptosis in CC.** (A-B) The expression of M16 was assayed in KMBC and RBE cells treated with erastin (20 μM) or RSL3 (1 μM) for 24 h by qPCR (A) and WB (B). (C) The proliferative effects of combination of knockdown M16 and liproxstatin or ferrostatin-1 in KMBC and RBE cells. (D-F) Mitochondria morphology (D), mitochondrial membrane potential (E) and intracellular ATP (F) were observed in KMBC cells transfected with si NC or si M16. (G-I) GSH (G), MDA (H), and Fe^2+^ levels (I) in M16-knockdown or M16-overexpressing KMBC cells were measured. (J-K) Lipid peroxide levels in M16-knockdown or M16-overexpressing KMBC cells were assayed by fluorescence microscopy (J) and flow cytometry (K) using C11-BODIPY. (L) The GSH effects of combination of knockdown M16 and liproxstatin (0.2 μM) and ferrostatin-1 (1 μM) in KMBC cells. The data are presented as the mean ± SD. * p < 0.05, ** p < 0.01, *** p < 0.005.

Given that the process of ferroptosis intricately involves oxidative stress, we gave more insight to the link between METTL16 and ferroptosis. We employed RSL3 and erastin to elicit ferroptosis in CC cells (Figure S2A) and observed that *METTL16* was significantly decreased during this process (Figure 4A and B), meaning that METTL16 is related to ferroptosis. Furthermore, we employed liproxstatin and ferrostatin-1 to inhibit ferroptosis in KMBC and RBE cells and found that METTL16 knockdown slows cell proliferation which is rescued by liproxstatin or ferrostatin-1 (Figure 4C), suggesting that METTL16 knockdown promotes ferroptosis in CC. Morphologically, ferroptosis has characteristic pathological changes, such as shrinkage of mitochondria, decrease or vanishing of mitochondrial cristae 14. Then, we observed changes in subcellular organelles in METTL16-knockdown KMBC and RBE cells by TEM and found that mitochondria became smaller and cristae decreased in METTL16-knockdown cells (Figure 4D, Figure S2B). In light of the fact that MMP and intracellular ATP produced mainly by mitochondria are closely related to mitochondrial cristae, the MMP and ATP were measured. The results showed that METTL16 knockdown decreased MMP and ATP, while METTL16 overexpression increased MMP and ATP (Figure 4E and F, Figure S2C). These findings verified that METTL16 knockdown elicits ferroptosis in CC. Biochemically, ferroptosis results from Fe^2+^ accumulation and LPO. Next, intracellular Fe^2+^ and metabolic product of LPO, such as GSH, MDA and LPO, were examined 28-30. The results showed that METTL16 knockdown decreased intracellular GSH levels but increased intracellular MDA, Fe^2+^ and LPO levels in KMBC and RBE cells. Conversely, METTL16 overexpression elevated intracellular GSH levels but decreased intracellular MDA, Fe^2+^ and LPO levels (Figure 4G-K, Figure S2D-H). In addition, the effects of METTL16 knockdown on GSH was revised by liproxstatin and ferrostatin-1 in KMBC cells (Figure 4L). In conclusion, these observations indicate that METTL16 suppresses ferroptosis in CC*.*

### ATF4 is a key target of METTL16 and its stability is regulated by METTL16 via m^6^A modification

**Figure 5:**
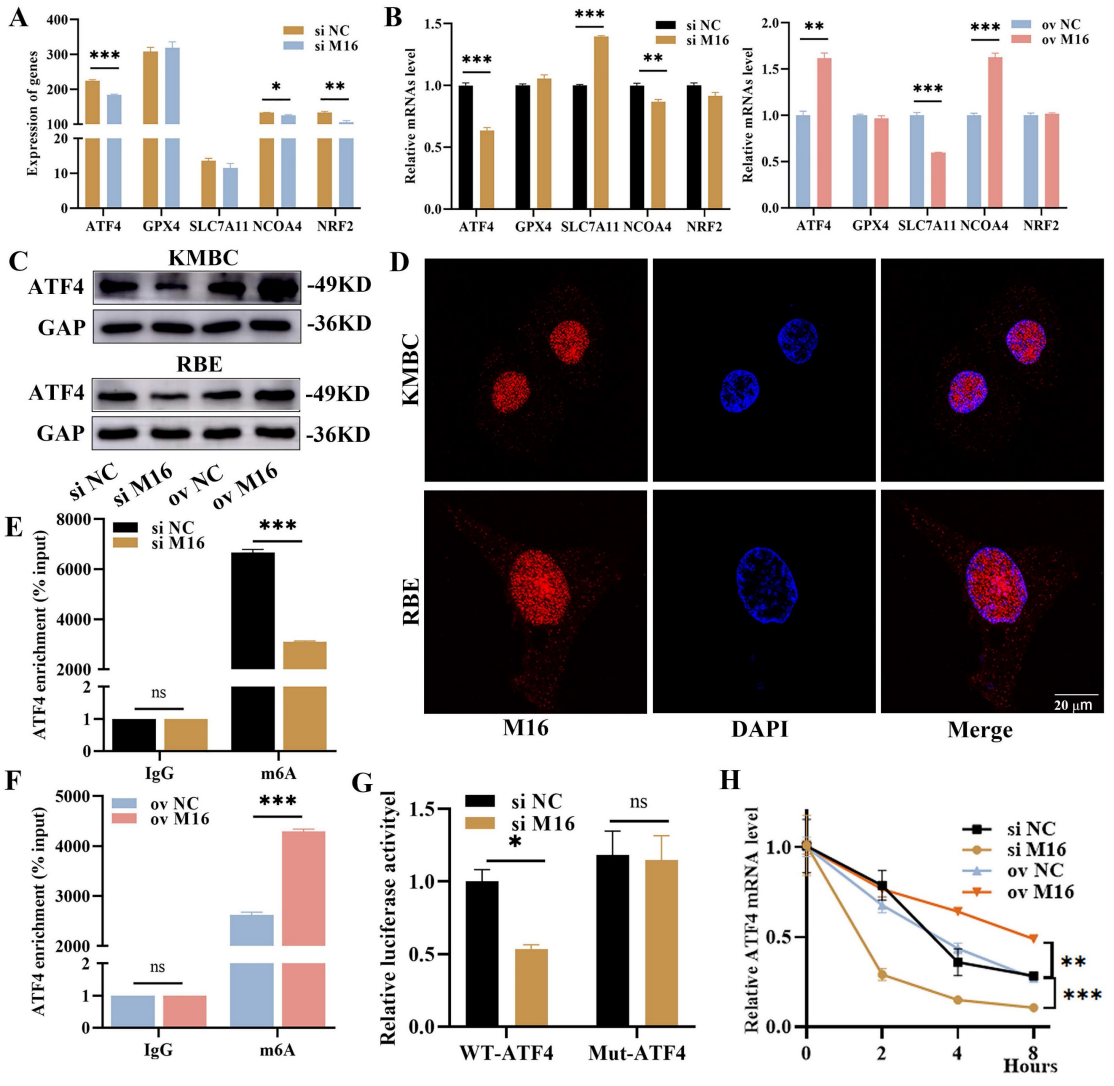
** M16 enhances ATF4 expression and stability.** (A) The expression of ferroptosis-related genes from RNA-sequencing. (B) The expression of ferroptosis-related genes in M16-knockdown or M16-overexpressing KMBC cells was confirmed by qPCR. (C) WB showed that the protein of ATF4 in M16-knockdown or M16-overexpressing KMBC and RBE cells. (D) The location of M16 in KMBC and EBE cells was shown by CLSM. (E-F) The m6A methylation level of ATF4 in KMBC cells transfected with si M16 (E), M16 plasmids (F) and controls. (G) Relative luciferase activity of ATF4-Wt and ATF4-Mut in M16-knockdown KMBC cells. (H) ATF4 mRNA levels in M16-knockdown and M16-overexpressing KMBC cells treated with actinomycin D. The data are presented as the mean ± SD. * p < 0.05, ** p < 0.01, *** p < 0.005.

Previous studies have shown that ferroptosis is regulated by several key genes, including NCOA4, ATF4, GPX4, SLC7A11 and NRF2. Among these genes, NCOA4 promotes ferroptosis, while the others inhibit ferroptosis 14. To detail the mechanism in ferroptosis regulated by METTL16, we reviewed the RNA-sequencing results and discovered that METTL16 knockdown reduced the expression of *ATF4*, *NCOA4* and *NRF2* (Figure 5A). Afterward, we further verified the impacts of METTL16 on these genes when knockdown and overexpression METTL16 in KMBC and RBE cells. In KMBC cells, METTL16 knockdown decreased the *NCOA4* and *ATF4* but elevated the *SLC7A11*, and METTL16 overexpression elevated the *ATF4* and *NCOA4* but reduced the *SLC7A11* (Figure 5B). In RBE cells, METTL16 knockdown reduced the *ATF4* and *NRF2* but elevated *SLC7A11*, and METTL16 overexpression increased the *ATF4*, *NCOA4* and *NRF2* (Figure S3A). These results showed that only ATF4 is consistently influenced by METTL16. Furthermore, WB revealed that METTL16 knockdown reduced ATF4 which was elevated after METTL16 overexpression *in vitro* (Figure 5C). In parallel, our analysis of GSE107943, GSE76297 and GSE132305 revealed a positive correlation between *METTL16* and *ATF4* (Figure S3B). We also knockdown other key m^6^A-reated genes, like METTL3, METTL14, METTL16, WTAP, ALKBH5, FTO, and found that other genes did not produce similar significant effects on ATF4 mRNA as METTL16 (Figure S3C). Thus, these findings confirm that ATF4 is a major regulatory target of METTL16.

The way that METTL16 exerts functions hinges on its location in cells. In the nucleus, METTL16 works in m^6^A-dependent manner, while in the cytoplasm, it does in an m^6^A-independent manner 11, 31. In CC tissues, KMBC and RBE cells, METTL16 was mainly localized in the nucleus (Figure 5D), suggesting that METTL16 takes effect primarily through m^6^A modification. Therefore, we postulated that ATF4 undergoes METTL16-mediated m^6^A modification and performed SRAMP analysis. The results manifested that there are three m^6^A modification sites on *ATF4* mRNA, among which the second appeared to be the most likely (Figure S3D). This suggested that METTL16 may interpose the m6A modification of ATF4 mRNA, as evidenced by MeRIP (Figure S3E). Notably, METTL16 knockdown reduced the m^6^A level of *ATF4*, while METTL16 overexpression increased the m^6^A level of *ATF4* (Figure 5E and F). Subsequently, dual-luciferase reporters were performed to further confirm the m^6^A site on ATF4 (Figure S3F). The results revealed that METTL16 knockdown significantly reduced the firefly luciferase in wild-type ATF4. However, METTL16 knockdown had no impact on the renilla luciferase activity from wild-type to mutant ATF4 (Figure 5G). Thus, the m^6^A modification of ATF4 is under the control of METTL16. It is widely acknowledged that m^6^A modification primarily regulates mRNA stability, leading to changes of mRNA expression 32. We performed RNA stability experiments and found that METTL16 knockdown decreased *ATF4* stability, whereas METTL16 overexpression increased ATF4 stability (Figure 5H, Figure S3G). Overall, these results suggest that METTL16 promotes expression and stability of ATF4 through m^6^A modification.

### ATF4 promotes CC progress and rescues the effect of METTL16 in CC

**Figure 6:**
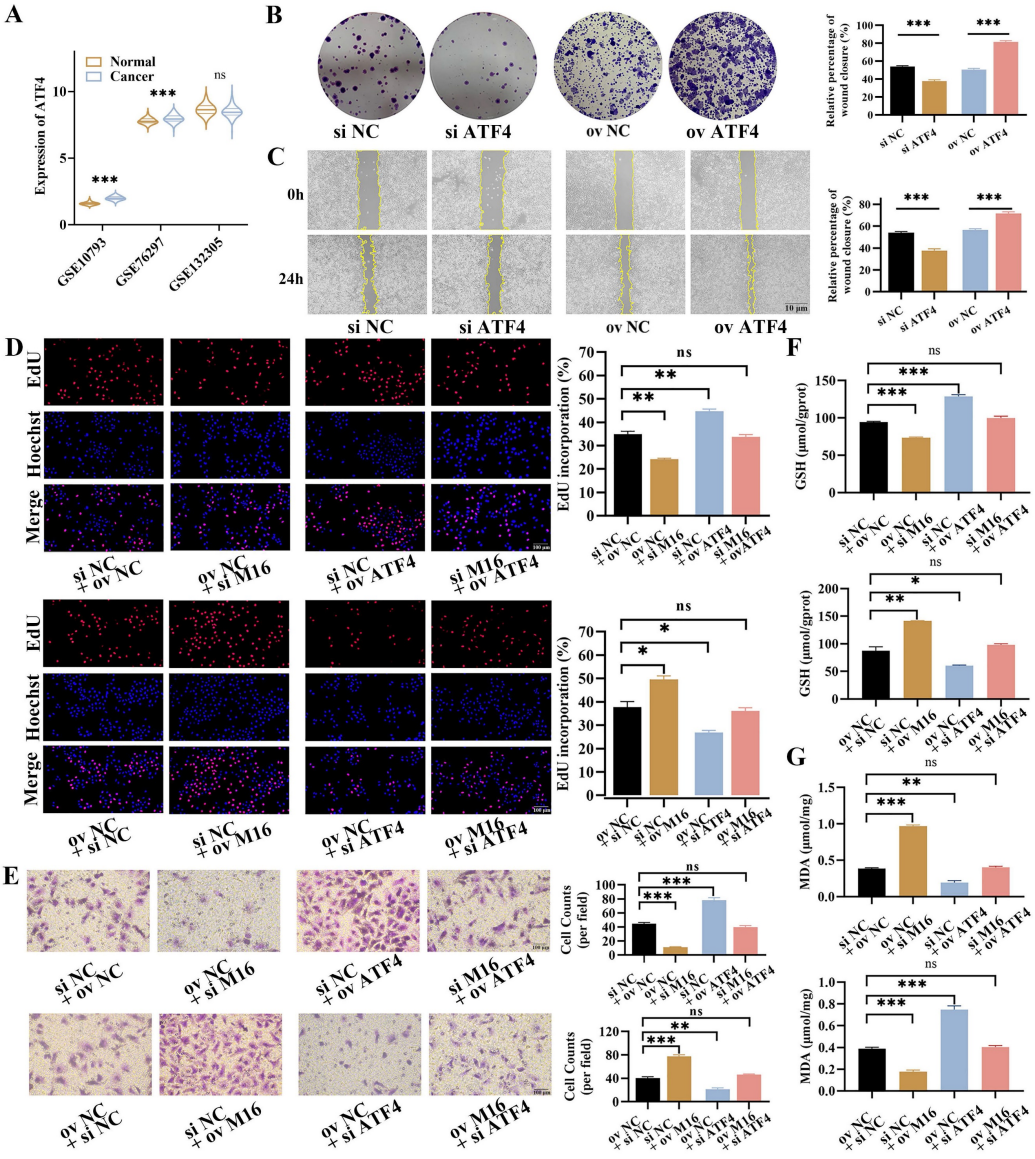
** ATF4 promotes the growth and rescues the effects of M16 in CC.** (A) The expression of ATF4 in GSE107943, GSE76297 and GSE132305. (B-C) The proliferation and migration of ATF4-knockdown or ATF4-overexpressing KMBC cells were investigated by colony formation assays (B) and wound healing assays (C). (D-E) ATF4 knockdown/overexpression rescued the M16-overexpressing/ knockdown effect on proliferation (D) and migration (E) in KMBC cells. (F-G) ATF4 knockdown/overexpression reversed the M16-overexpressing/ knockdown effect on GSH (F) and MDA (G) levels in KMBC cells. The data are presented as the mean ± SD. * p < 0.05, ** p < 0.01, *** p < 0.005.

Then, the role of ATF4 in CC was further explored. GEO analysis showed that *ATF4* was upregulated in CC tissues (2/3 datasets), which was in line with HPA, indicating that ATF4 may play an oncogenic role in CC (Figure 6A, Figure S4A). In order to testify this notion, ATF4 knockdown and overexpression were carried out and verified in KMBC and RBE cells (Figure S4B and C). The EdU assays showed that ATF4 knockdown reduced proliferation in KMBC and RBE cells, whereas ATF4 overexpression increased proliferation (Figure S4D). These findings were in line with the results of colony formation (Figure 6B, Figure S4E). Furthermore, The Transwell assays manifested ATF4 knockdown reduced the migration of KMBC and RBE cells, while ATF4 overexpression increased migration (Figure S4F). Similarly, the migrative functions of ATF4 were further confirmed by wound-healing assays (Figure 6C, Figure S4G). To further verify the functions of ATF4 in METTL16-induced effects in CC, rescue experiments were performed. In EdU assays, ATF4 overexpression restored the reduced proliferative capacity caused by METTL16 knockdown in KMBC and RBE cells, whereas ATF4 knockdown abolished the increased proliferative capacity resulting from METTL16 overexpression (Figure 6D, Figure S5A). Similar migration results were obtained in Transwell assays (Figure 6E, Figure S5B). Together, these findings show that ATF4 facilitate the growth and revises METTL16-induced progression in CC.

As known, ATF4 is a key regulatory gene in ferroptosis, which could inhibit ferroptosis and enhances tumor growth 22, 33. Here, we found that ATF4 knockdown decreased intracellular GSH levels but elevated intracellular MDA levels, while ATF4 overexpression increased intracellular GSH levels but decreased intracellular MDA levels in KMBC and RBE cells (Figure S5C and D). In rescue assays, ATF4 overexpression reversed the METTL16 knockdown-induced changes in intracellular GSH and MDA levels in KMBC and RBE cells, while ATF4 knockdown restored the METTL16 overexpression-induced changes in intracellular GSH and MDA levels (Figure 6F and G, Figure S5E and F). Collectively, these observations demonstrate that ATF4 is responsible for METTL16-mediated regulation of ferroptosis in CC.

### METTL16 inhibits ferroptosis in CC by ATF4 in mice

**Figure 7:**
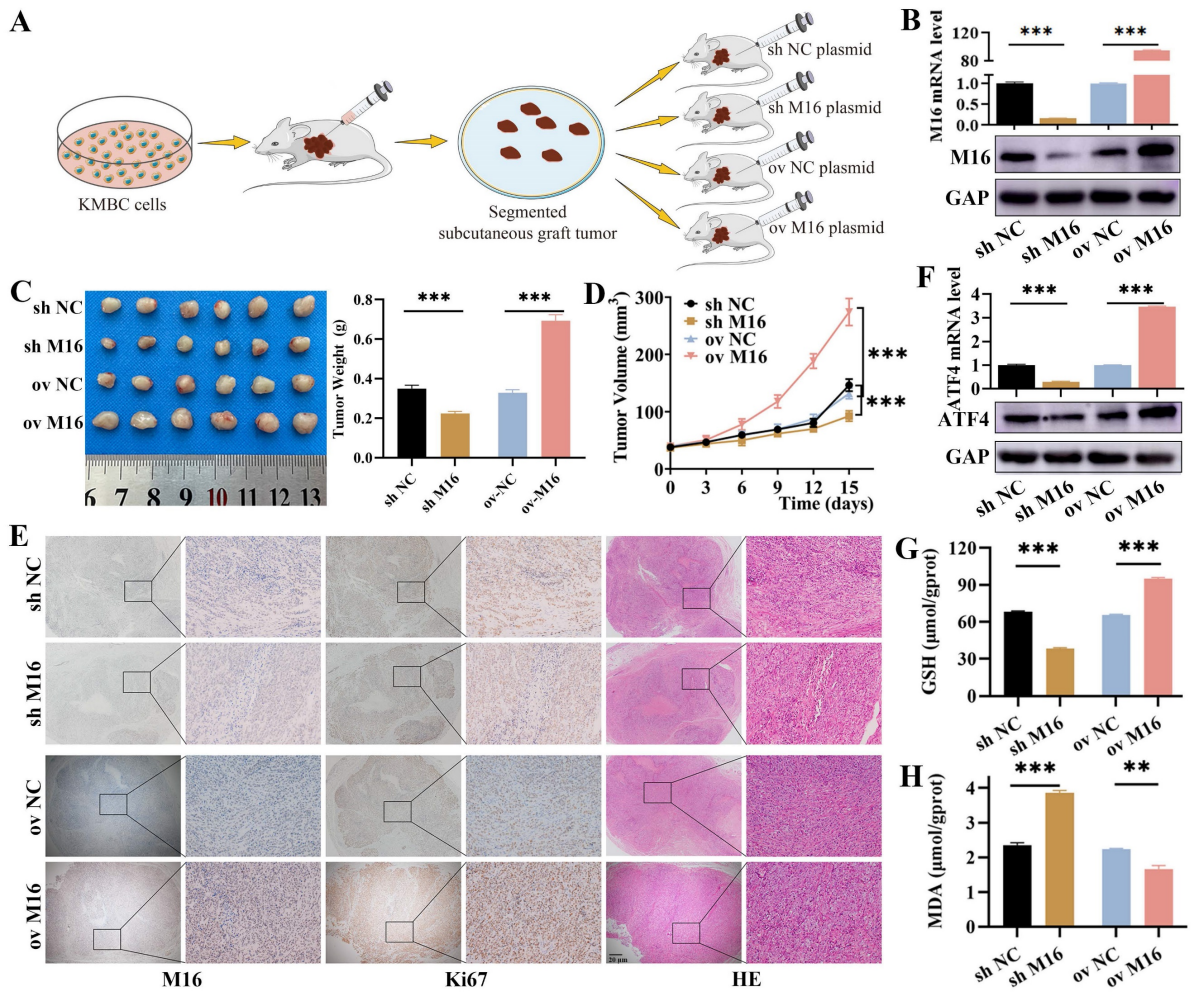
** M16 inhibits ferroptosis in CC via ATF4 *in vivo*.** (A) M16 overexpression or knockdown xenograft model in nude mice. (B) Xenograft tumors with M16 knockdown or overexpression were confirmed by qPCR and WB. (C-D) The tumor weight (C) and volume (D) of the xenografts were measured. (E) Xenograft tumors were stained for M16, Ki-67, and HE. (F) qPCR and WB showed that the expression of ATF4 in the xenograft tumors with M16 knockdown or overexpression. (G-H) GSH (G) and MDA (H) levels in the xenograft tumors were measured. The data are presented as the mean ± SD. ** p < 0.01, *** p < 0.005.

Based on the results from *in vitro* studies, nude mouse xenograft models (Figure 7A) with METTL16 knockdown and overexpression plasmids were constructed to validate the role of METTL16 in CC *in vivo*, and the effectiveness of METTL16 overexpression and knockdown was confirmed by qPCR, WB and IHC (Figure 7B and E). As shown, METTL16 knockdown reduced tumor growth and weight, whereas METTL16 overexpression increased tumor growth and weight (Figure 7C and D). Furthermore, we used Ki-67 and HE staining to assess whether cell proliferation and necrosis were affected by METTL16 knockdown and overexpression. Our results revealed that METTL16 knockdown reduced proliferation but increased necrosis, while METTL16 overexpression enhanced proliferation but suppressed necrosis (Figure 7E). Regarding to ferroptosis, we found METTL16 knockdown decreased the expression of ATF4, while METTL16 overexpression increased the expression of ATF4 (Figure 7F). Then, we tested indicator of ferroptosis in tissues and found that METTL16 knockdown decreased GSH levels but increased MDA. Conversely, METTL16 overexpression elevated GSH levels but decreased MDA (Figure 7G and H). Taken together, these results indicate that METTL16 promotes progression and ferroptosis in CC *in vivo*.

## Discussion

In recent years, there have been great advancements in targeted drugs and immunotherapy, but the prognosis of patients with CC remains poor. Thus, more attention should be paid to new therapeutics. In this study, we found the potential of METTL16 as a treatment for CC and revealed a novel mechanism for improving anti-tumor effects by inducing ferroptosis. Specifically, METTL16 is upregulated and enhances ATF4 mRNA stability and expression via m^6^A modification, further counteracting ferroptosis and promoting the growth of CC**.**

Among the approximate 150 post-transcriptional RNA modifications that have been identified, m^6^A modifications are the most prevalent 34, 35. m^6^A modifications play oncogenic or tumor-suppressive roles in cancer by regulating RNA splicing, translocation, degradation, stability, and translation. As a new m^6^A methyltransferase, METTL16 not only regulates MAT2A expression in response to yield of s-adenosyl methionine (SAM) that is an essential cofactor for methylation, but also binds to MAT2A to form a METTL16-RNA complex, which consequently drives downstream RNA methylation 36-38. Here, we found that METTL16 is highly expressed in CC and correlated with poor prognosis. Whatmore, METTL16 overexpression enhanced the migration and proliferation of KMBC and RBE cells, while METTL16 knockdown inhibited these biological behaviors, similar to the results *in vivo*. These data manifested that METTL16 has tumor-promoting impacts on CC, which is consistent with previous reports in hepatocellular carcinoma, colorectal carcinoma, gastric cancer and lung cancer 38-41. In contrast, METTL16 suppresses cell proliferation through the p21 pathway in pancreatic adenocarcinoma 42. These contradictory functions in different cancers may have occurred due to tissue specificity and cancer specificity.

Metabolic reprogramming is a central hallmark of cancers, and METTL16 has a great impact on metabolic reprogramming, such as branched chain amino acid metabolism and glucose metabolism 41, 43. We also found that METTL16 is mainly associated with oxidative stress, as confirmed by measuring ROS levels. Ferroptosis is activated by oxidative stress that results from glutathione depletion, hemin, and/or reactive lipid species 44, 45. Thereafter, we explored whether METTL16 affects the process of ferroptosis. Morphologically, METTL16 knockdown caused mitochondria to become smaller with cristae disappearing, and decreased the MMP, indicating that METTL16 acts against ferroptosis in CC. Mechanistically, METTL16 knockdown decreased intracellular GSH levels and increased MDA, Fe^2+^ and LPO levels, similar to the findings in breast cancer 46. METTL16 overexpression elevated intracellular GSH levels and reduced MDA, Fe^2+^ and LPO levels. These results suggest that METTL16 facilitates the progression of CC by suppressing ferroptosis.

In response to the mechanism of METTL16 in regulating ferroptosis, previous studies have shown three different ways to improve METTL16-mediated resistance to ferroptosis: (i) SAM, ascended by METTL16, ultimately gives rise to cysteine via the trans-sulfuration pathway, which recures lipid peroxidation and impedes 47,48; (ii) SAM also increases H3K4me3 modification on the promoter of ACSL3 and subsequently upregulates its expression, protecting against ferroptosis 49; and (iii) METTL16 promotes the expression and stability of GPX4 to further confront ferroptosis 46. Here, another novel way from our research was that METTL16 relies on ATF4 to inhibit ferroptosis in an m^6^A-dependent manner. First, we found that METTL16 is primarily localized in the nucleus and to a lesser extent in the cytoplasm. In the nucleus, METTL16 is involved in the m^6^A modification of target RNAs, while in the cytoplasm, METTL16 masters cell fate through translation in an m^6^A-independent manner 11, 31. These data mirrored that METTL16 exerts its effect mainly via m^6^A modification. Next, according to RNA-sequencing, ATF4 was confirmed to be another downstream target of METTL16 and to be modified by METTL16-mediated m^6^A methylation, as confirmed by MeRIP and dual-luciferase reporter assays. Furthermore, RNA stability assays showed that the stability of ATF4 mRNA is significantly reduced after METTL16 knockdown, but that increased after METTL16 overexpression. Overall, these data provide strong evidence that METTL16 promotes the expression and stability of ATF4 to curb ferroptosis via m^6^A modification.

ATF4 is an important regulator in response to hypoxia or amino acid restriction 50, 51. Most of its target genes are involved in various salvage pathways that promote cell survival, but others prepare the cell for death 52. In hepatocellular carcinoma, renal cell carcinoma and pancreatic ductal adenocarcinoma, ATF4 suppresses ferroptosis and promotes cell proliferation through the ATF4/SLC7A11 and ATF4/HSPA5/GPX4 axes 22, 23, 33. Conversely, ATF4 facilitates apoptosis and exerts anti-tumor effects via the ATF4/CHOP axis in the treatment of multiple myeloma with aspirin 53. As to CC, the function of ATF4 is not yet fully understood. Our study first showed that ATF4 is highly expressed in CC and accelerates migration and proliferation by inhibiting ferroptosis. Moreover, ATF4 knockdown abolished the effects of METTL16 overexpression on proliferation and ferroptosis, and ATF4 overexpression reversed the effects of METTL16 knockdown. Based on these findings, it is confirmed that METTL16/ATF4 performs important functions on ferroptosis in CC. As known, ATF4 is a common upstream regulator of both SLC7A11 and GPX4 those inhibit ferroptosis by producing GSH and detoxifying lipid peroxidation, respectively 21-23. However, whether both SLC7A11 and GPX4 are affected by METTL16/ATF4 remains unknown, and further experiments are needed.

Our work elucidates the role of METTL16 in CC and its ability to inhibit ferroptosis. METTL16 is upregulated in CC tissues, conferring a poor prognosis for patients. Mechanistically, METTL16 promotes the expression and stability of ATF4, thereby suppressing ferroptosis in CC and ultimately accelerating tumor growth. These findings provide valuable insights into METTL16-mediated m6A modifications in CC as well as effective therapeutic strategies for CC.

## Supplementary Material

Supplementary figures and tables.
