# Supplementary material for: METTL16 suppresses ferroptosis in cholangiocarcinoma by promoting ATF4 via m6A modification
Source: Int J Biol Sci. 2025 Jan 1;21(1):189–203. doi: 10.7150/ijbs.97886 (PMC11667817; doi:10.7150/ijbs.97886)
Supplement: Supplementary file 1 — Supplementary figures and tables. [file ijbsv21p0189s1.pdf]

Table S1. The target sequence of siRNAs

siRNAs	Sequence
si ALKBH5	GAACTACTGGCGCAAGTCATA
si ATF4	CCTGAAAGATTTGATAGAA
si FTO	GACCTTCCTCAAGCTCAATGA
si M16 #1	GATGGATGCTCTTAAAGAA
si M16 #2	CCACCAAGTAAGCGAAGAA
si METTL14	GATTGCAGCACCTCGATCATT
si METTL3	GCACTTCAGACGAATTATCAA
si WTAP	GAGATGCAAGAGTGTACTACT

Table S2. The Antibodies for WB, IHC and MeRIP

Antibody Name	Brand
ATF4	D4B8, Cell Signaling Technology, Boston, MA, USA
CoraLite594-conjugated Goat Anti-Rabbit IgG(H+L)	SA00013-4, Proteintech, Wuhan, China
GAPDH	10494-1-AP, Proteintech, Wuhan, China
HRP Goat Anti-Rabbit IgG (H+L)	AS014, ABclonal, Wuhan, China
IgG	PP64-10-KC, Millipore, Darmstadt, Germany
Ki-67	GB111499-100, Servicebio, Wuhan, China
m6A	202 003, Synaptic Systems, Goettingen, Germany
METTL16	ab313743, Abcam, Cambridge, USA

Table S3. The sequence of primers

**Gene**	**Sequence (5'-3')**
ALKBH5-F	TCAAGCCTATTCGGGTGTCG
ALKBH5-R	GGGTGCATCTAATCTTGTCTTCC
ATF4-F	CCCTTCACCTTCTTACAACCTC
ATF4-R	TGCCCAGCTCTAAACTAAAGGA
ATF4-F (for MeRIP)	GACCTTCTGACCACGTTGGA
ATF4-R (for MeRIP)	AAGAAGGTGAAGGGGGCAAC
FTO-F	CGAGTGGCAGAGTGCTCAAC
FTO-R	TCAGCCACTCAAACTCGACC
GAPDH-F	GGAGCGAGATCCCTCCAAAAT
GAPDH-R	GGCTGTTGTCATACTTCTCATGG
GPX4-F	GAGGCAAGACCGAAGTAAACTAC
GPX4-R	CCGAACTGGTTACACGGGAA
METTL14-F	TTGGACCTTGGAAGAGTGTGT
METTL14-R	TGAATGAAGTCCCCGTCTGTG
METTL16-F	AGGGAGTAAACTCACGAAATCCT
METTL16-R	AACCCCTTGTATGCGAAGCTC
METTL3-F	TTGTCTCCAACCTTCCGTAGT
METTL3-R	CCAGATCAGAGAGGTGGTGTAG
NCOA4-F	GAGGTGTAGTGATGCACGGAG
NCOA4-R	GACGGCTTATGCAACTGTGAA
NRF2-F	AGGTTGCCCACATTCCCAAA
NRF2-R	AGTGACTGAAACGTAGCCGA
SLC7A11-F	GGTCCATTACCAGCTTTTGTACG
SLC7A11-R	AATGTAGCGTCCAAATGCCAG
WTAP-F	ACTGGCCTAAGAGAGTCTGAAG
WTAP-R	GTTGCTAGTCGCATTACAAGGA

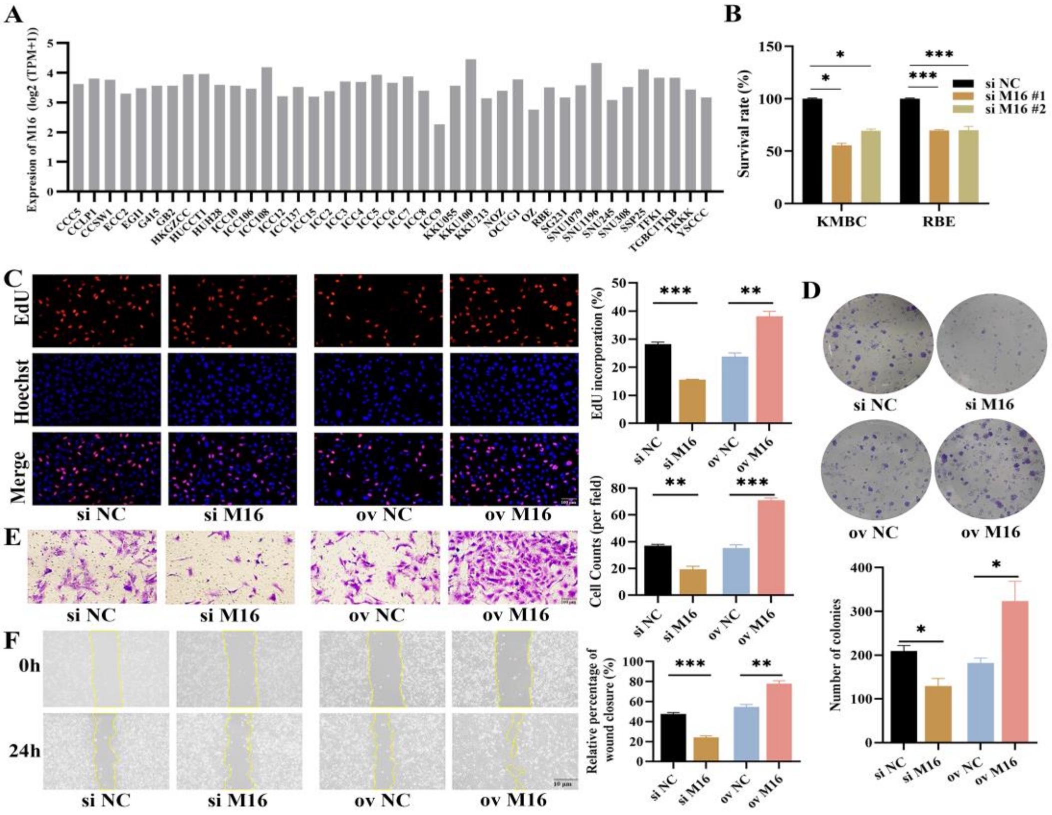

Figure S1. M16 promotes CC progression. (A) Expression of M16 in CC cell from DepMap. (B) The survival rate of knockdown M16 in KMBC and RBE cells was measured by CCK8. (C-F) The proliferation and migration of M16-knockdown or M16-overexpressing RBE cells were tested by EdU assays (C), colony formation assays (D), Transwell assays (E) and wound-healing assays (F). The data are presented as the mean ± SD. * $p < 0.05$, ** $p < 0.01$, *** $p < 0.005$.

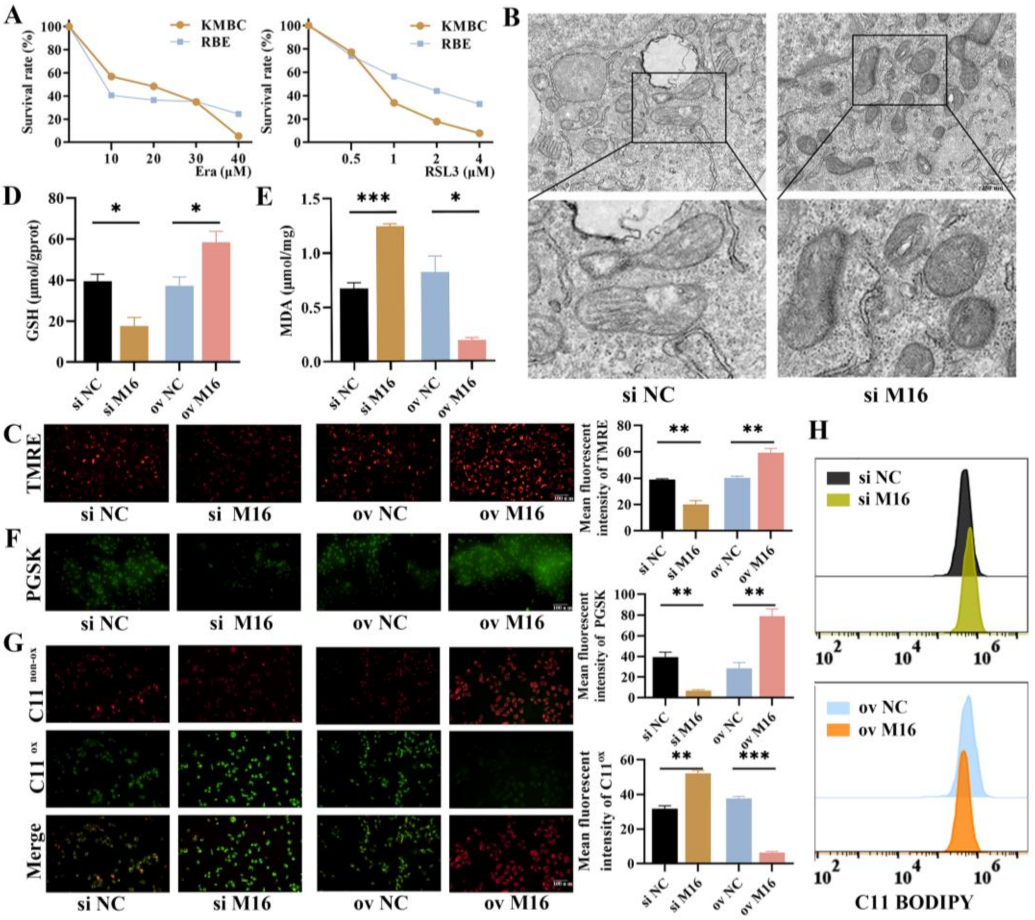

Figure S2. M16 inhibits ferroptosis in CC. (A) Cell viability was tested in KMBC and RBE cells after treatment with erastin or RSL3 for 24 h. (B-C) Mitochondria morphology (B) and mitochondrial membrane potential (C) were observed in RBE cells transfected with si NC or si M16. (D-F) GSH (D), MDA (E) and Fe^{2+} (F) levels in M16-knockdown or M16-overexpressing RBE cells. (G-H) Lipid peroxide levels in M16-knockdown or M16-overexpressing RBE cells were measured by fluorescence microscopy (G) and flow cytometry (H) using C11-BODIPY. The data are presented as the mean ± SD. * *p < 0.05*, ** *p < 0.01*, *** *p < 0.005.*

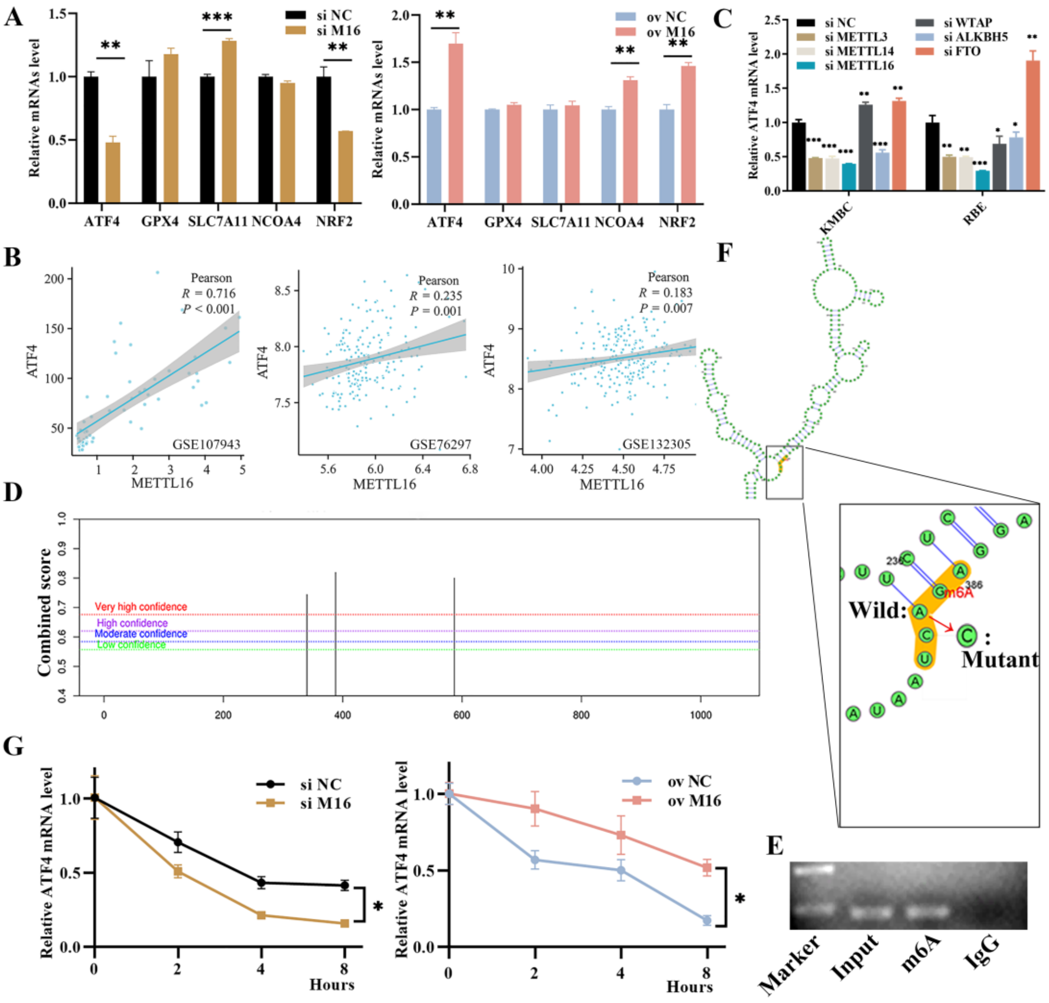

Figure S3. M16 enhances ATF4 expression and stability. (A) The expression of ferroptosis-related genes in M16-knockdown or M16-overexpressing RBE cells was measured by qPCR. (B) Positive correlation of M16 expression and ATF4 expression in CC tissues from GSE107943, GSE76297 and GSE132305. (C) The expression of ATF4 in KMBC and RBE cells after other m6A-related genes knockdown was measured by qPCR. (D) The methylated sites on ATF4 mRNA were predicted by SRAMP. (E) The m6A methylation of ATF4 in KMBC cells was confirmed by nucleic acid electrophoresis. (F) The 388 methylated sites on ATF4 mRNA were used to construct wild-type and mutant plasmids. (G) ATF4 mRNA levels in M16-knockdown and M16-overexpressing RBE cells treated with actinomycin D. The data are presented as the mean ± SD. * $p < 0.05$, ** $p < 0.01$.

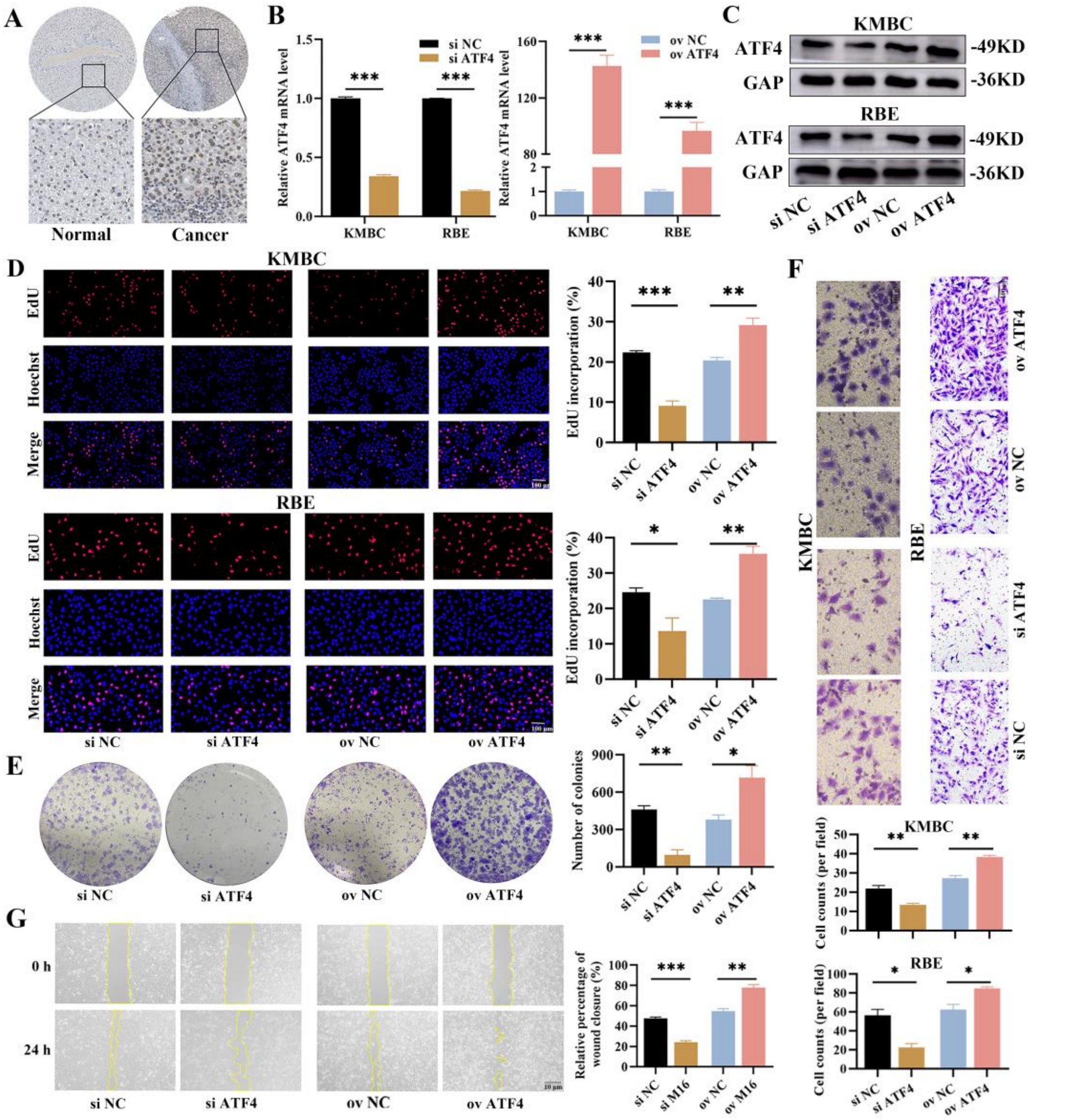

Figure S4. ATF4 enhances the tumorigenicity in CC. (A) The images of IHC staining for ATF4 in CC and normal tissues from HPA. (B-C) ATF4 knockdown or overexpression in KMBC and RBE cells was confirmed by qPCR (B) and WB (C). (D, F) The proliferation and migration of ATF4-knockdown or ATF4-overexpressing KMBC and RBE cells were measured by EdU assays (D) and Transwell assays (F). (E, G) The proliferation and migration of ATF4-knockdown or ATF4-overexpressing RBE cells were evaluated by colony formation assays (E) and wound healing assays (G). The data are presented as the mean ± SD. * $p < 0.05$, ** $p < 0.01$, *** $p < 0.005$.

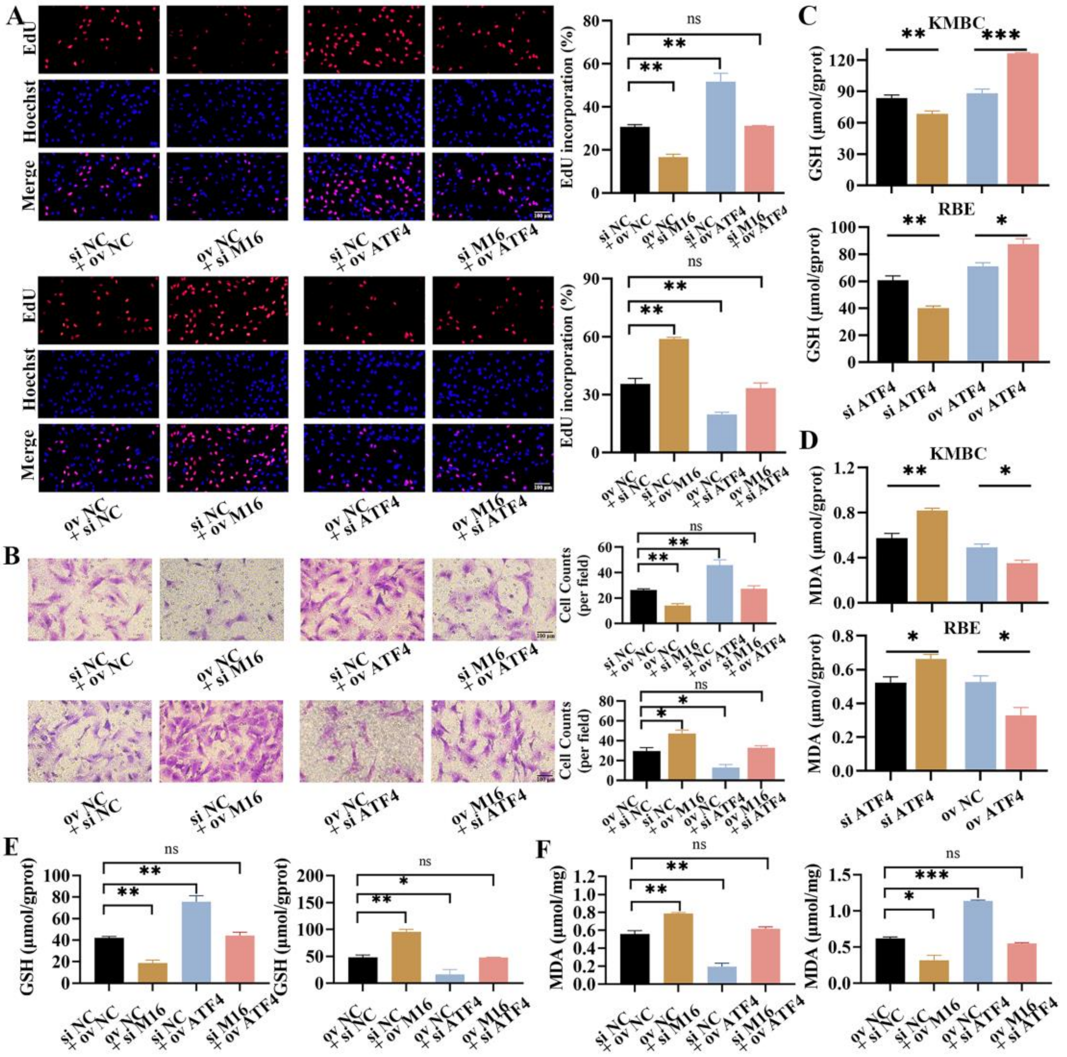

Figure S5. ATF4 rescues the effects of M16. (A-B) ATF4 knockdown/overexpression rescued the M16-overexpressing/ knockdown effect on proliferation and migration in RBE cells. (C-D) GSH (C) and MDA (D) levels in M16-knockdown or M16-overexpressing KMBC cells were measured. (E-F) ATF4 knockdown/overexpression reversed the M16-overexpressing/ knockdown effect on GSH (E) and MDA (F) levels in RBE cells. The data are presented as the mean ± SD. ** $p < 0.05$, ** $p < 0.01$, *** $p < 0.005$.*
